# A Reliable Pipeline Leak Detection Method Using Acoustic Emission with Time Difference of Arrival and Kolmogorov–Smirnov Test

**DOI:** 10.3390/s23239296

**Published:** 2023-11-21

**Authors:** Duc-Thuan Nguyen, Tuan-Khai Nguyen, Zahoor Ahmad, Jong-Myon Kim

**Affiliations:** 1 Department of Electrical, Electronic and Computer Engineering, University of Ulsan, Ulsan 44610, Republic of Korea; ducthuan@mail.ulsan.ac.kr (D.-T.N.); khaint@mail.ulsan.ac.kr (T.-K.N.); zahooruou@mail.ulsan.ac.kr (Z.A.); 2 PD Technology Co., Ltd., Ulsan 44610, Republic of Korea

**Keywords:** leak detection, pipeline systems, acoustic emission, time difference of arrival, cross-correlation, Kolmogorov–Smirnov test

## Abstract

This paper proposes a novel and reliable leak-detection method for pipeline systems based on acoustic emission (AE) signals. The proposed method analyzes signals from two AE sensors installed on the pipeline to detect leaks located between these two sensors. Firstly, the raw AE signals are preprocessed using empirical mode decomposition. The time difference of arrival (TDOA) is then extracted as a statistical feature of the two AE signals. The state of the pipeline (leakage/normal) is determined through comparing the statistical distribution of the TDOA of the current state with the prior normal state. Specifically, the two-sample Kolmogorov–Smirnov (K–S) test is applied to compare the statistical distribution of the TDOA feature for leak and non-leak scenarios. The K–S test statistic value in this context functions as a leakage indicator. A new criterion called leak sensitivity is introduced to evaluate and compare the performance of leak detection methods. Extensive experiments were conducted using an industrial pipeline system, and the results demonstrate the excellence of the proposed method in leak detection. Compared to traditional feature-based indicators, our approach achieves a significantly higher performance in leak detection.

## 1. Introduction

Pipelines are vital industrial infrastructure, as they play a crucial role in fluid transportation in industry [1]. They are also one of the most popular transportation modes in the world due to their affordability, safety, and economic benefits [2]. However, pipeline leaks can occur due to factors such as material corrosion, fatigue cracks, earthquakes, material defects, and environmental influences [3,4]. Leakage can cause significant waste of resources, resulting in economic losses as well as posing potential risks of environmental pollution [5]. Some leakage incidents have been reported in [6] with the loss of over USD 200,000 in China and hundreds of deaths and injuries in Mexico due to the leakage in diesel and petroleum pipelines. Therefore, early leak state detection is extremely important and urgent to confirm the stability and the reliability of pipeline systems. It must be emphasized that leak state detection is the task of identifying the presence of a leak in a pipeline without considering its location. Therefore, leak state detection does not involve localization, which is inherently challenging and time-consuming to accomplish.

In general, there are various approaches to detect pipeline leaks, such as nondestructive testing methods like ultrasonic testing, acoustic emission, pressure and flow monitoring, or optical fiber sensing [7,8]. Among these techniques, AE technology is widely preferred in the industry due to its capability in real-time response, high sensitivity, and ease of installation [9,10,11]. The origins of AE technology can be traced back to its inception in 1950 through the pioneering efforts of Joseph Kaiser [12]. Throughout the 1950s and ‘60s, scientists extensively explored the basics of acoustic emission, designed specialized equipment tailored for AE, and studied the AE patterns exhibited by various materials [12]. Specifically, AE is the spontaneous release of elastic energy by a material when it undergoes deformation [13]. Once a leak occurs in the pipeline, AE is generated by the turbulence, cavitation, or high-velocity flow associated with the leakage [13]. These AE signals are created and propagated instantaneously along the pipeline, making them responsive and sensitive to leaks [14]. These advantages enable AE-based methods to quickly identify the presence of leaks on pipelines. Given these advantages, AE technology is employed in this study for pipeline leak state detection.

In recent years, research on leak state detection has focused on two main approaches: signal processing (SP)-based and artificial intelligence (AI)-based methods [15,16]. In the first approach, statistical features of the signal are manually extracted based on expert knowledge and understanding. These features are then used as representative of the pipeline’s condition, based on their changes to identify the presence of leaks. Wang et al. [17] obtained distinctive AE features in the time domain and then employed principal component analysis to reduce the features’ dimension. A support vector data descriptor was utilized as a detector afterward. Wang et al. [18] involved analyzing the frequency change through the amplitude of frequencies related to leaks to detect leakage in fluid pipelines. Similarly, Xiao et al. [19] collected several different characteristics from the AE signal in both the time and frequency domains, namely, the mean, root mean square, kurtosis, skewness, mean/median frequency, and frequency slope. The leak-related features were ranked and selected using the Kullback–Leibler distance, and some typical machine learning models were utilized as detectors.

On the other hand, in recent years, AI-based approaches for pipeline leak state detection have gained popularity due to their superior ability to learn from data by themselves. Bae et al. [20] created pattern images for ensemble learning by reflecting leakage signal properties in the time and frequency domains, then used them with state-of-the-art residual networks. Zhou et al. [21] developed a new ensemble transfer learning model for pipeline leak state detection and localization based on a one-dimensional convolutional neural network called TL1DCNN. Ahmad et al. [22] used the continuous wavelet transform to create acoustic spectrograms from time series acoustic emission signals. They were fed through a convolutional autoencoder and convolutional neural network to extract and integrate features before being fed into a shallow artificial neural network to detect leaks. Though these AI-based models can achieve high performance on pipeline leak state detection, they still have the following limitations. Firstly, AI methods require a large amount of leakage pipeline labeled data for training models [23]. In industry, it is challenging to obtain such data as it is time-consuming and risky when leaks happen. Secondly, AI methods are uninterpretable, i.e., technicians cannot understand why the model made a specific prediction, leading to a lack of reliability [24].

**Figure 1:**
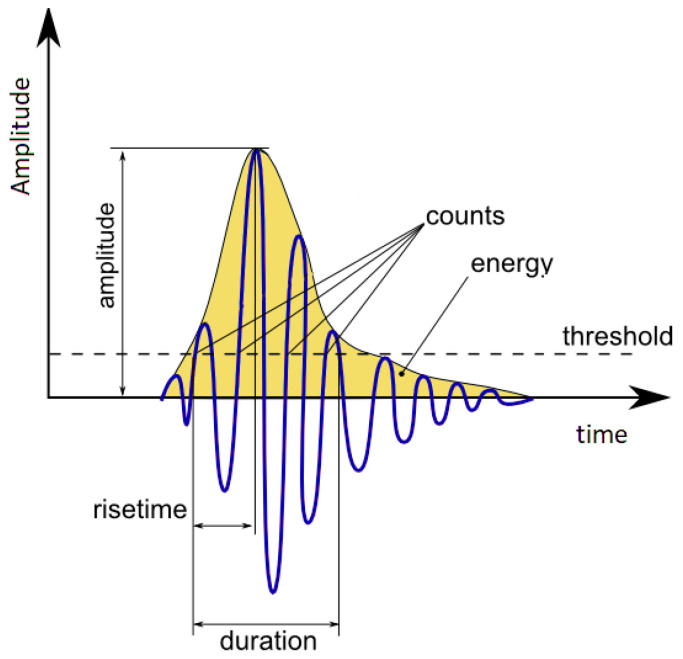
Conventional AE hit-based features.

In order to address the limitations associated with AI-based techniques, which require a huge amount of labeled data, this study concentrated on employing signal processing approaches for the identification of pipeline leaks. Nevertheless, the signal processing methods discussed earlier typically rely on extracting features from a single AE sensor, which can be susceptible to external acoustic emission (AE) interference, such as rain or impact [25]. Moreover, conventional statistical features like the mean, root mean square, variance, and kurtosis from only one sensor are also susceptible to background noise and acoustic propagation attenuation, resulting in a heightened false alarm rate during leak state detection [26]. As for AE-based parameters such as the rise time, peak amplitude, duration, counts, and energy, they highly depend on the chosen threshold, making it challenging to determine (see Figure 1). Furthermore, relying on these parameters can easily lead to confusion with AE noise, such as rain. These are motivations for us to propose a more reliable and accurate method for leak state detection in pipeline systems.

In this work, we propose a method to detect leaks between the two AE sensors based on processing AE signals generated on the pipeline between these two sensors. From our observations, in the normal state (under the normal working condition without leakage), acoustic emissions appear randomly on the pipe as noise. When a leak occurs, the source of acoustic emission events is highly concentrated on the leak location. Hence, the source of AE events might be a potential feature for identifying the appearance of leaks. The time difference of arrival (TDOA) is a well-known technique for AE source localization and the relationship between TDOA and AE source location is linear [27]. Specifically, TDOA calculates the time difference between the arrivals of signals to determine the differences in their travel distances based on their velocities. Subsequently, the source location is accurately determined. The TDOA can be challenging to apply in determining the source of AE because it relies on the wave velocity. However, TDOA can still somehow indicate the source position. Therefore, the main idea is that we will monitor the change in the TDOA value distribution to detect leaks. Accordingly, the raw AE signals will be preprocessed first using empirical mode decomposition (EMD) to remove irrelevant components as well as improve the signal-to-noise ratio [28]. The TDOA then is extracted from the two improved AE signals as a statistical feature representing the pipe state (normal/leak). 

Afterward, the two-sample K–S test, a popular statistical distribution test, is performed to compare the distribution of TDOA between the current state and the normal one. The larger the statistic value obtained, the larger the distribution difference, representing the greater probability of leaks appearing. The K–S test is highly valuable for leak state detection when a reference dataset representing normal conditions is available. It is non-parametric, making it suitable for complex TDOA distributions associated with leaks. The K–S test is sensitive to differences in distribution shape and other characteristics, making it effective in detecting subtle changes in TDOA features caused by leaks. Its distribution-free nature and ease of implementation add to its practicality in real-world leak state detection applications. Based on the conducted analyses, the utilization of the K–S test is the most appropriate choice for this study, as it aligns well with the characteristics of the dataset within the research context.

The main contribution of this research is summarized as follows:

- We proposed a novel and reliable indicator for pipeline leak state detection based on AE signals using the time difference of arrival feature and the two-sample K–S test.
- Verification and evaluation experiments were conducted using a custom industrial pipeline system for effectiveness and robustness of the proposed method.

The rest of this paper is organized as follows. Section 2 of the paper introduces the foundational background concepts, while the third section outlines the proposed method. In Section 4, a case study is conducted using a real pipeline system, accompanied by the results and discussion. Finally, Section 5 serves as the conclusion.

## 2. Background Concepts

### 2.1. Empirical Mode Decomposition (EMD)

One of the main advantages of using EMD [29] for leak state detection is its ability to handle non-stationary and non-linear characteristics of the signals often encountered in leak state detection scenarios [30]. Another advantage of EMD is its ability to handle noise and artifacts in the signal [31]. By decomposing the signal into intrinsic mode functions (IMFs) and a residue, EMD allows for the selective removal or analysis of specific components, making it a valuable tool for denoising and feature-extraction tasks [32]. In this work, EMD is implemented to preprocess the raw AE signals. By doing this, the signal-to-noise ratio of the signals will be increased and reveal AE patterns clearly, aiding in the accurate estimation of TDOA.

The principal of EMD is demonstrated as follows. Given an original signal $x\left(t\right)$, in the first step, the upper and lower envelope of $x\left(t\right)$ are generated by connecting all the local maxima and minima of the signal using the cubic spline line method. In the second step, the first component $h_{1}\left(t\right)$ of $x\left(t\right)$ is determined using the difference between $x\left(t\right)$ and the mean m(t) of the upper and lower envelopes:

(1) $$h_{1}\left(t\right)=x\left(t\right)-m(t)$$

An IMF must satisfy the following two conditions: (i) the number of extremes must equal the number of zero-crossings or differ at most by one, and (ii) the mean value of the upper and lower envelopes must be zero. Ideally, $h_{1}\left(t\right)$ should satisfy the definition of an IMF. If not, $h_{1}\left(t\right)$ is treated as an original signal and the process is repeated to obtain $h_{2}\left(t\right)$:

(2) $$h_{2}\left(t\right)=h_{1}\left(t\right)-m_{1}(t)$$

where $m_{2}(t)$ is the mean of the upper and lower envelopes of $h_{1}\left(t\right)$. The process is repeated k times until we obtain the first IMF $h_{k}\left(t\right)$, referred to as the first-order IMF, denoted as $c_{1}\left(t\right)$. After that, this IMF will be subtracted from the original signal to obtain the residual $r_{1}\left(t\right)$:

(3) $$r_{1}\left(t\right)=x\left(t\right)-c_{1}(t)$$

This residual is considered a new original signal, and the process of extracting the IMF is repeated. The process only stops if, at that point, the residual is a monotonic signal.

### 2.2. Time Difference of Arrival

The time difference of arrival (TDOA) method is a technique used for the localization of sources in signal processing [33]. It works by measuring the time delay between the arrival of a signal at multiple sensors or receivers. This method has been widely used in various applications such as radar systems, acoustic localization, and wireless sensor networks [34]. In this work, TDOA is extracted from two AE sensors as a statistical feature through one of the most popular techniques, called cross-correlation. For two signals $f\left(t\right)$ and $g\left(t\right)$, the cross-correlation is defined as [35]:

(4) $$X\left(\tau \right)=\int_{-\infty }^{\infty }f\left(t\right)g\left(t+\tau \right)dt$$

where τ is called the lag time. The cross-correlation is a function of the similarity between two signals over the time shift (lag time) between them. The lag time at which the signals are most similar is considered the TDOA of those signals.

### 2.3. Two-Sample Kolmogorov–Smirnov Test

The Kolmogorov–Smirnov test (K–S test) is a statistical method for determining if two continuous or discontinuous one-dimensional probability distributions are equal [36]. The K–S test used to compare the distribution of two samples is called the two-sample K–S test. In the two-sample K–S test, the empirical cumulative distribution function (eCDF) of n independent and identically distributed observations x is defined as [37]:

(5) $$F\left(x\right)=\frac{number\;of\;elements\leq x\;}{n}$$

To compare two samples, their empirical distribution functions $F\left(x\right)$ and $G\left(x\right)$ are first determined. Then, the hypotheses are defined as follows: Null Hypothesis ($H_{0}$): $F\left(x\right)=G(x)$, and Alternative Hypothesis ($H_{1}$): $F\left(x\right)\neq G(x)$. The Kolmogorov–Smirnov statistic value D is originally defined as follows [38]:

(6) $$D=\underset{x}{\max}\left|F\left(x\right)-G\left(x\right)\right|$$

The $H_{0}$ is rejected when the test statistic is higher than a pre-defined threshold. In this work, the two-sample K–S test is utilized to detect leaks by comparing the eCDFs of the TDOA features obtained from the pipeline to ones collected from known healthy conditions. The greater the differences between the eCDFs, the more they reveal the presence of leaks, corresponding to higher values of D.

## 3. Proposed Method

**Figure 2:**
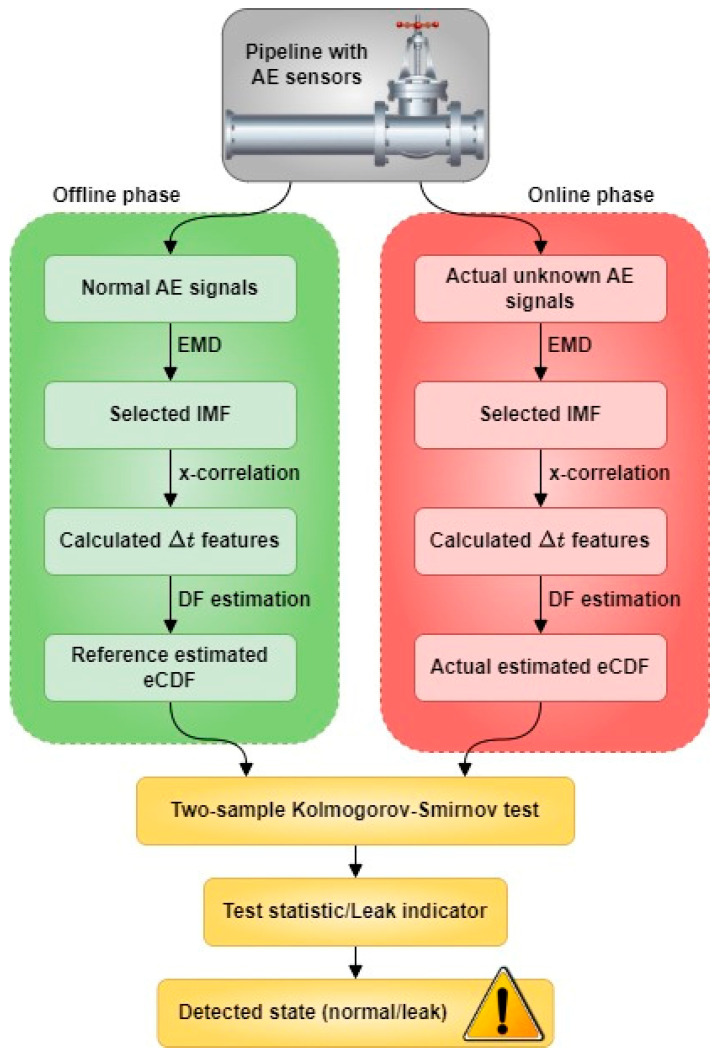
The flowchart of the proposed method.

The flowchart for pipeline leak state detection following the proposed method is shown in Figure 2.

**Figure 3:**
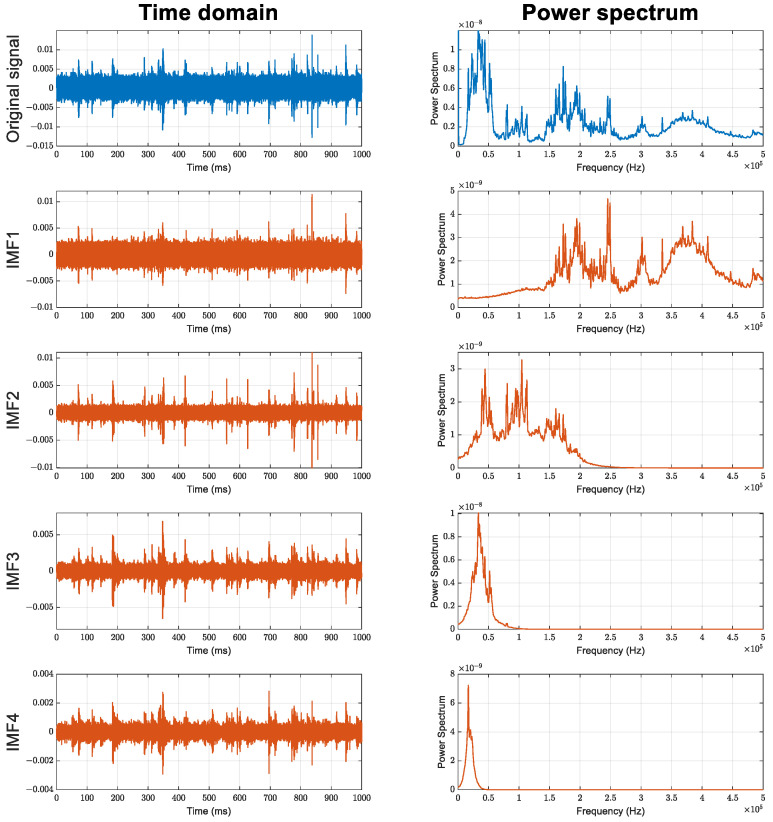
Illustration of a raw AE signal and its IMFs in time domain and power spectrum.

The proposed procedure is divided into two phases: the offline phase and the online phase, both consisting of similar steps. In each phase, the goal is to estimate the empirical cumulative distribution function (eCDF) corresponding to each state (normal/leak) and then use them as inputs for the K–S test. The detailed steps for leak state detection implementation are sequentially performed as follows:

- Step 1: AE signals from the two AE sensors are collected from the pipeline.
- Step 2: EMD decomposition is implemented to decompose the raw AE signals into intrinsic mode functions (IMFs). Figure 3 illustrates the time-domain signal and the power spectrum of a raw AE signal along with its IMFs. Since low-order IMFs contain high-frequency components, they effectively represent the AE signals [39]. Experimental results show that the first-order IMF usually contains high-frequency noise (over 150 kHz) and should be disregarded [40]. Thus, in this step, the second-order IMF is selected for further processing, as it contains useful and less noisy AE signals.
- Step 3: TDOA (Δt) of the two AE signals is calculated using the cross-correlation (x-correlation) technique. To avoid randomness, Δt should be calculated on various scales of signal length and should be selected from several lag times with the highest correlation instead of only one. Hence, the AE signals are divided into multiple segments with three different lengths based on the estimated length of AE events. Then, three lag times corresponding to the highest correlations are selected to represent the Δt.
- Step 4: The eCDFs of the Δt values obtained in step 3 are estimated (DF estimation). For normal AE signals in the offline phase, the resulting eCDFs are referred to as reference estimated eCDF. For actual AE signals (with unknown states) in the online phase, the resulting eCDFs are considered actual estimated eCDFs.
- Step 5: A two-sample K–S test is conducted to examine the similarity between actual and reference estimated eCDFs. The statistical value obtained from the test is considered as an indication of the pipeline leakage state. A higher index suggests a higher probability of pipeline leakage.

## 4. Case Study

### 4.1. Experimental Setup

**Figure 4:**
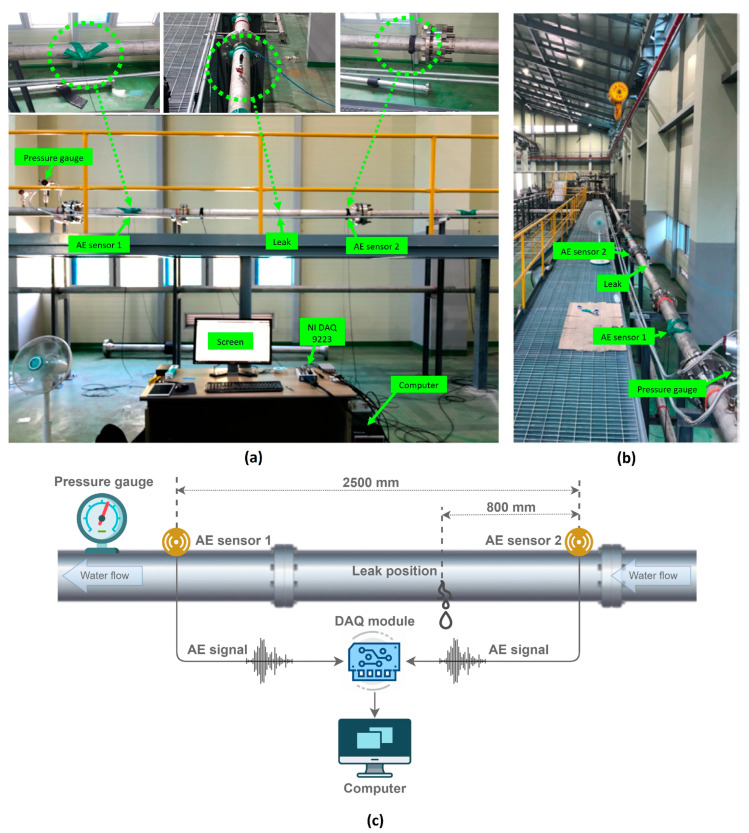
Experimental testbed setup for data acquisition: (**a**) front view, (**b**) side view, (**c**) schematic.

To assess the effectiveness of the suggested approach in detecting leaks, we established a practical test environment in an actual industrial fluid pipeline. The testbed where the AE signals acquired is a part of a larger network of industrial pipelines, depicted in Figure 4a,b.

In this testbed system, there are multiple straight steel pipelines connected to each other by flanges. According to Wichaidit et al. [41], flanges do impact the acoustic wave velocity on the pipe surface, causing reflections and diffractions and altering wave propagation patterns and velocities. While this affects the wave arrival time, it is invariant, and as our research focuses on changes in Δt distribution, it does not impede leak state detection. Two MITRAS R15I-AST sensors (see Table 1 for the specification) are attached to the pipeline surface and spaced 2500 mm apart, as depicted in Figure 4c. These sensors are mounted onto the pipeline using industrial tape to ensure the contact and fixed position. Due to the different surface geometry, we apply a specialized gel suggested by the manufacturer to enhance the contact between the sensor and the pipe. In this study, we only use 2 sensors to save costs because it is the minimum required to detect leaks in a one-dimensional material pipeline. In cases where the leak location is unknown in advance, we can deploy more sensors and execute the proposed algorithm on each pair of adjacent sensors. The NI DAQ-9223 data acquisition module from National Instruments (NI) is used to convert AE signals from sensors to digital signals and transfer them to the computer via a high-speed USB interface. The computer used is a personal computer with a one-terabyte hard drive for data storage. We developed custom software based on the Python language and NI’s interface library to control the data acquisition process. Prior to data acquisition, the pencil leak break test is conducted to determine if calibration was necessary, to ensure the ability to detect the AE signal from the sensors.

To simulate leaks on the pipeline, a specialized tool was directly welded onto the pipe at 800 mm from one sensor, as shown in Figure 4c. This tool essentially functions as a valve with a perforated core used to simulate leaks. We used this tool to change the size of the leak by replacing its core. The data collection scenario involved creating various contexts for water release at different sizes of leaks (0.3, 0.5, and 1.0 mm) at different pressure levels (7, 13, and 18 bars). In total, nine different scenarios were created by combining the three leak sizes and three pressure levels.

For each size of leak, the data acquisition process involved starting with the leak closed and a pressure level of 7 bars. The data recorded in the first 100 s are considered normal AE signals. Then, the leak was activated in the next 100 s and remained unchanged throughout the acquisition. The leak was then deactivated, and the same steps were repeated for pressure levels of 13 and 18 bars. Deactivating the leak before each pressure-level change ensured stable fluid flow for normal working conditions at each pressure level. All the AE signals were collected at a sampling frequency of 1 MHz. To facilitate the analysis, we estimated the duration of AE events and subsequently divided the collected data files into segmented data with appropriate lengths of 10, 50, and 100 μs, which correspond to 10,000, 50,000, and 100,000 data points, respectively. To enhance the data and preserve the interconnections between segments, a 75% overlap is applied.

Our goal is to validate the leak state detection capability of the proposed method in various working conditions of pipelines. Therefore, our experiments include calculating leak indicators for data collected before and after the leak is activated in nine introduced data scenarios. By monitoring changes in the indicator, we can detect the presence of a leak evidently. To evaluate and compare the leak state detection capability of different methods, we introduce a criterion called leak sensitivity S, which is defined as follows:

(7) $$S=\left|E{\left[I\left(t_{l}\right)-I\left(t_{n}\right)\right]}_{t_{l}>t_{n}}\right|$$

where E is the expectation, I(t) is the indicator value at a specific time t, and $t_{n}$ and $t_{l}$ are the times when the leak has not yet occurred and has occurred, respectively. The leak sensitivity represents the ability of the leak indicator to change significantly before and after a leak occurs. The higher the value of S, the greater the likelihood of detecting a leak.

### 4.2. Results and Discussion

**Figure 5:**
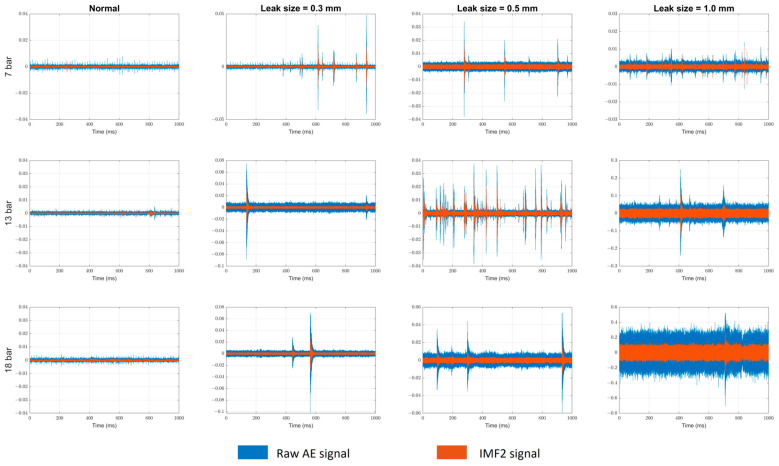
Raw AE signals of sensor 2 and the corresponding IMF2 at multiple pressure levels.

This section provides a comprehensive analysis of the experimental results, accompanied by discussions. The raw acoustic emission (AE) signals obtained from sensor 2 and their corresponding IMF2 at different pressure levels of the pipe are presented in Figure 5.

**Figure 6:**
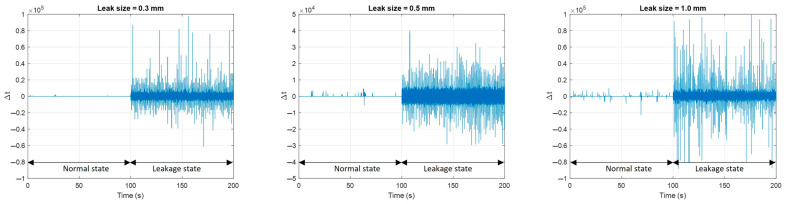
Δt features of pipeline at normal and leakage states under 7 bar pressure.

The Δt features in this study are calculated by analyzing the IMF2s of two different sensors using cross-correlation techniques. To minimize the impact of random variations, three specific lag times corresponding to the three highest correlation values between the two recorded signals are considered. An illustrative example of the Δt features of a pipeline experiencing leakage at the pressure level of 7 bar is depicted in Figure 6. It is evident from the graph that when a leak occurs, there is a significant fluctuation in the Δt values; however, the overall trend cannot be easily observed. Hence, it is not appropriate to directly use Δt as a reliable indicator of leakage, and instead, its probability distribution needs to be employed for assessment.

**Figure 7:**
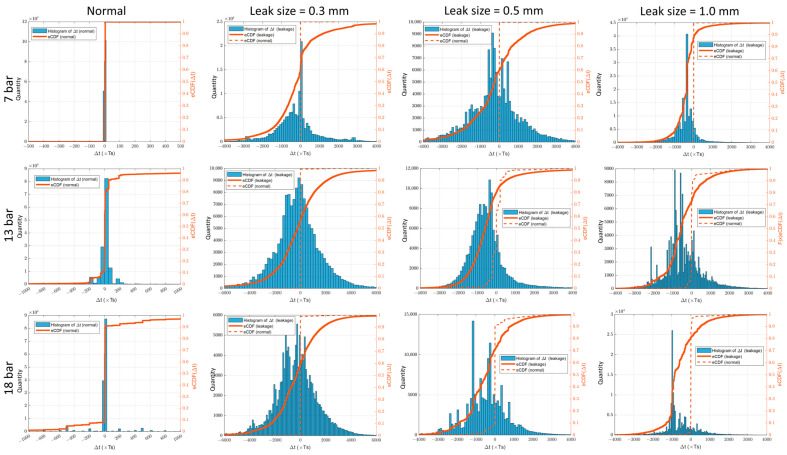
Histograms of the calculated Δt features and corresponding eCDF in various scenarios.

Figure 7 presents histograms depicting the calculated Δt features and corresponding empirical cumulative distribution functions in different scenarios (where $T_{S}=10^{-6}$ s). A comparison between the normal state and various leakage states shows clear differences in the distribution of Δt, particularly in terms of mean and standard deviation/variance. In the normal state, the mean value of Δt is approximately 0, and the standard deviation/variance is negligible. Conversely, the leakage states exhibit a shift from the mean and significantly larger values for standard deviation/variance. This finding remains consistent across all three pressure levels, suggesting the potential to effectively distinguish leakage from the normal state. The shift in mean value can be attributed to Δt transitioning from 0 to a value closer to the position of the leak. The increased standard deviation/variance is a result of the inherent randomness in TDOA calculations. Additionally, the TDOA value is influenced by the acoustic emission (AE) wave velocity, which fluctuates depending on the propagation mode of the wave [13].

The distribution of Δt affects the shape of the corresponding eCDF. In the normal state, the eCDF takes the form of a Heaviside step function, while in the leakage state it exhibits a smooth variation. Moreover, the probability density of Δt is predominantly concentrated on the left side of the vertical axis due to the order of signals in TDOA calculation. Consequently, the eCDF in the leakage state intersects the vertical axis at a point above 0.5. This observation indicates that the statistical value obtained from the K–S test will likely be relatively large (greater than 0.5), allowing for a clear differentiation between the leakage state and the normal state, which should yield a value lower than 0.5.

**Figure 8:**
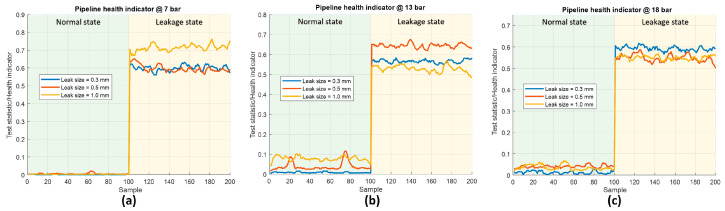
The proposed leak indicator for pipeline working under pressures of (**a**) 7 bar, (**b**) 13 bar, and (**c**) 18 bar.

Figure 8a–c depict the leak indicator proposed in this study, represented by the test statistic derived from the two-sample Kolmogorov–Smirnov (K–S) test for the Δt samples obtained every second. This indicator reveals the disparities in the distribution of the Δt attributes extracted from the actual pipe under normal and leak conditions. Regardless of the circumstances, the indicator value remains around 0.1 or below when the pipe is in a normal state, while it exceeds 0.5 when a leak occurs. Consequently, users can clearly differentiate between these two states. Moreover, the indicator exhibits stability when the system remains in a particular state, thus ensuring the stability of the state and strengthening the reliability of leak state detection. Notably, when a state change occurs at the 100th second, the indicator promptly reflects this alteration, highlighting its capacity to monitor state transitions promptly and accurately.

To highlight the enhanced capabilities of the proposed method in comparison to traditional methods, a comparative analysis was conducted. Traditional methods typically employ well-known statistical features, such as the mean, root mean square (RMS), standard deviation (std. dev.), variance, or kurtosis, as indicators of the system’s state. In this study, these five features were utilized for comparison with the proposed method. 

**Figure 9:**
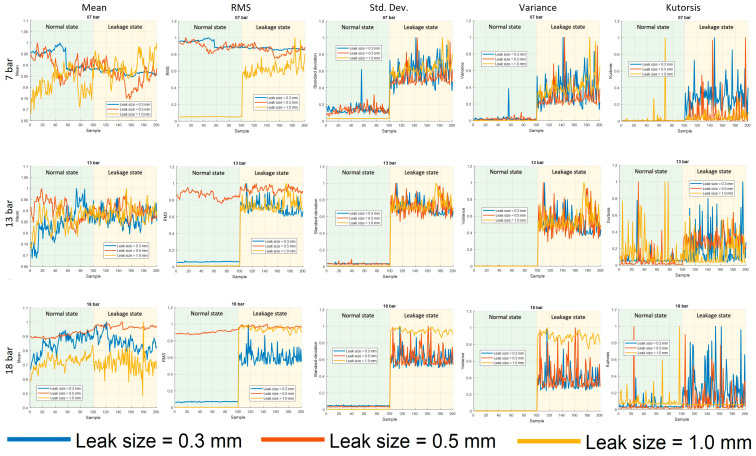
Representation of indicators based on traditional features.

Figure 9 presents the representation of indicators based on traditional features. Specifically, the indicators mean, RMS, and kurtosis are unable to distinguish leakage states effectively, as there is no significant change in indicator values when leakage occurs. Moreover, if a slight change is observed, it cannot be considered a dependable indicator unless it happens in various situations. Conversely, features such as std. dev. and variance exhibit noticeable changes in both value and probability distribution when leakage occurs. Hence, they can be directly or indirectly utilized as indicators through statistical testing. One should be cautious while using these features as they are prone to noise interference.

As for the qualitative aspect, it can be argued that the proposed method for pipeline leak state detection possesses greater reliability compared to statistical features. To bolster this assertion, a quantitative analysis is undertaken using the leak sensitivity criterion as the basis for assessing leak state detection. To ensure fairness, the values of the examined indicators are normalized within the range of 0 and 1 for computational purposes. To demonstrate this, Table 2 presents a comparison of the leak sensitivity index of different indicators at different pressure levels.

In terms of the leak sensitivity value, the proposed method consistently outperforms all other indicators. For a 7-bar pressure, the sensitivity value is 0.93 to 0.95, for a 13-bar pressure it is 0.82 to 0.94, and for an 18-bar pressure it is 0.89 to 0.94. Furthermore, the standard deviation and variance exhibit a leak sensitivity higher than 0.26 and remain relatively high across different scenarios. Conversely, kurtosis, mean, and RMS prove ineffective in detecting leaks, as they display low sensitivity or instability. In summary, comparisons confirm the superiority of the proposed method in leak state detection when compared to traditional methods. This accomplishment can be attributed, at least in part, to the introduction of the TDOA feature and the refinements made to the calculations.

## 5. Conclusions

This paper presented a novel approach to detect leaks in pipeline systems. Early detection of leaks in pipelines is an urgent task in the industry because it limits resource loss and ensures public safety. The proposed method suggested utilizing two AE sensors to capture AE signals occurring within the pipeline. By analyzing TDOA extracted from the two preprocessed signals, leaks between the two sensors could be identified. To achieve this, we applied the two-sample K–S test to compare the probability distribution of the extracted TDOA with the reference distribution representing the normal state. The test statistic served as an indicator for determining whether the state is normal or experiencing a leak, with higher values indicating a higher likelihood of a leak. 

Verification experiments were conducted using data collected from an actual pipeline system. The experimental outcomes clearly demonstrated the effectiveness of the proposed method in accurately differentiating between normal and leak states and capturing the moment of transition between them. Moreover, the proposed method outperformed traditional approaches based on statistical measures such as the mean, RMS, standard deviation, variance, and kurtosis, not only in terms of qualitative assessment but also in quantitative leak sensitivity. This achievement is due to the proposed method using the TDOA feature, which is less affected by noise and wave attenuation. In the future, further experiments will be conducted to evaluate the proposed method’s resistance to noise.

## Figures and Tables

**Table 1:** The specification of the R15I-AST sensors from MITRAS (Newark, NJ, USA).

Parameter	Value	Unit
Peak sensitivity	109	dB
Operating frequency	50 to 400	kHz
Resonant frequency	75	kHz
Directionality	±1.5	dB
Operating temperature	−35 to 75	°C

**Table 2:** Leak sensitivity comparison between examined indicators.

Pressure Level	Leak Size	Mean	RMS	Std. Dev.	Variance	Kurtosis	Proposed Method
7 bar	0.3 mm	0.06	0.06	0.46	0.35	0.29	0.95
	0.5 mm	0.05	0.05	0.38	0.26	0.09	0.94
	1.0 mm	0.08	0.58	0.60	0.40	0.02	0.93
13 bar	0.3 mm	0.02	0.65	0.68	0.51	0.14	0.94
	0.5 mm	0.01	0.07	0.69	0.53	0.25	0.89
	1.0 mm	0.03	0.73	0.73	0.55	0.03	0.82
18 bar	0.3 mm	0.01	0.54	0.56	0.37	0.20	0.94
	0.5 mm	0.06	0.07	0.60	0.41	0.10	0.89
	1.0 mm	0.01	0.94	0.94	0.89	0.04	0.90

## Data Availability

The data are available upon request.
